# Supplementary material for: The urgency of resuming disrupted dog rabies vaccination campaigns: a modeling and cost-effectiveness analysis
Source: Sci Rep. 2021 Jun 14;11:12476. doi: 10.1038/s41598-021-92067-5 (PMC8203735; doi:10.1038/s41598-021-92067-5)
Supplement: Supplementary file 2 — Supplementary Information 2. [file 41598_2021_92067_MOESM2_ESM.pdf]

APPENDIX – SUPPLEMENTAL METHODS AND RESULTS

The urgency of resuming disrupted dog rabies vaccination campaigns: a modeling and cost-effectiveness analysis

Amber Kunkel, Seonghye Jeon, Haim C Joseph, Pierre Dilius, Kelly Crowdis, Martin I. Meltzer, Ryan Wallace

Model Parameters and Initialization

Table S1 shows key parameters from the model. Parameters are separated into Ouest Department and the rest of Haiti, as the model was run separately for these two different regions. Additional rabies natural history parameters were the same as for previously published versions of the model (1, 2). The models listed here are those for the baseline scenario.

Table S1: Model parameters, baseline scenario

Variable	Value: Ouest	Value: Non-Ouest	Source	Notes
Program area	1,308 km^2	4,877 km^2		Excluding low-density or poorly connected areas; see notes below
Human population	3,794,146	4,993,365		Excluding low-density or poorly connected areas; see notes below
Human population density	2,900	1,024		Population/area; excluding low-density or poorly connected areas
Human birth rate (per 1,000 pop)	24		(3)	
Human life expectancy	64 years		(3)	
Human-to-Dog ratio at start of burn-in	20	10		See notes below
Spay/neuter	0%			Assumed
PEP coverage	85%	80%	(4)	94% of those with exposure to a confirmed rabid animal and 67% probable started vaccination after counseling
Ratio suspected rabies exposures: true rabies exposures (PEP efficiency)	5		(5)	10:1 is consistent with data from the United States and Haiti (Fig S2), but may not hold in an epidemic situation; values of 1-10x explored in sensitivity analyses
Average duration of immunity	2 years		(6-8)	Though vaccines used are licensed for 1 year only, this value seems conservative
Dog natural life expectancy	3 years		(9, 10)	This is if no rabies and no carrying capacity
Dog birth rate (per 1,000 dogs)	650			This corresponds to an observed average life expectancy of ~1.5 years at the end of the burn-in period
Rabies R_0 dog-to-dog	1.5		(11, 12)	
Dog-human transmission rate	$2.34*10^{-5}$		(7) (13)	This is the value used in (7); furthermore, the output numbers of human rabies deaths in Haiti are consistent with previous modeled estimates (13). Note this can be conceptualized as (average # humans bitten per rabid dog)/(human population density)
Community bite surveillance costs per case investigated	\$10.14 (USD)		(2)	
Cost per dog vaccinated	\$2.92 (USD)			Based on an evaluation of a vaccination campaign in Artibonite Department in 2017
Cost of post-exposure prophylaxis, per person	\$72.91 (USD)		(2)	

APPENDIX – SUPPLEMENTAL METHODS AND RESULTS

The urgency of resuming disrupted dog rabies vaccination campaigns: a modeling and cost-effectiveness analysis

Modified values of the dog birth rate, the dog natural life expectancy, and the R_0 for dog-dog transmission used in sensitivity analyses are shown in Table S2.

Table S2: Parameter values modified in sensitivity analyses.

	**Baseline scenario**	**"High turnover" scenario**	**"Low turnover" scenario**
Dog birth rate (per 1,000 dogs)	650	800	500
Dog natural life expectancy, years	3.0	2.5	3.5
R_0 dog to dog*	1.5	1.3	1.7

* R_0 is the average number of dogs infected with rabies per infectious (rabid) dog, in a situation where all other dogs are susceptible to infection.

In estimating human and dog population density, we aimed to include only those areas with density and connectivity high enough to maintain rabies transmission in the absence of isolated importation events (14, 15). To define low-density areas, we used a shapefile from the Gates Foundation containing polygons for inhabited areas (16). Using QGIS, we classified each polygon according to the department in which it was located (for polygons crossing department borders, we took the one with greatest overlap). We used the surface area of inhabited areas inside and outside of Ouest Department as our input areas.

We then calculated the population for each polygon based on population raster grids from (17) using the "zonal statistics" command. We used the calculated population of the inhabited areas inside and outside of Ouest Department as our input population values. We assumed a human-to-dog ratio of 20:1 (Ouest Department) and 10:1 (outside Ouest Department) in the absence of rabies. These values are consistent with estimates from other urban areas (14, 18), and were chosen to reflect the greater human density in Ouest Department. Surveys taken in 2014-2015 in 5 departments of Haiti showed human-to-dog ratios of 3-147 humans/dog. Excluding the outlier of 147, the mean estimates were 14.6 in Ouest and 9.7 outside Ouest. Surveys in 16 communities of Croix-des-Bouquets Commune, Ouest Department in 2016 ranged from 4-29 humans per dog.

The model was initialized by adding a small number of cases and running forward until a steady state is reached. In contrast to previous implementations of RabiesEcon, we assumed 5% annual vaccination coverage during this burn-in period, until 2005.

APPENDIX – SUPPLEMENTAL METHODS AND RESULTS

The urgency of resuming disrupted dog rabies vaccination campaigns: a modeling and cost-effectiveness analysis

Model Fit 2005-2020

The model-predicted number of reported cases and the number of rabies exposures for Haiti from 2005-2020 are roughly consistent with observed surveillance data. Though the trends do not fully match, surveillance efforts have been variable over time, and the results are consistently within an order of magnitude of the expected values. Table S3 shows the trends in human rabies deaths detected by surveillance compared to model estimates, as well as model estimates of human rabies exposures, under the baseline parameter set for the years 2005-2020.

Table S3: Model predicted reported rabies deaths, total rabies deaths, and total rabies exposures for 2005-2020, compared to surveillance data on rabies deaths per year.

Year[1]	Assumed Dog Vaccination Coverage	Surveillance-Detected deaths to rabies (% of model-estimated total)	Model estimate: reported[2] human rabies deaths = 10% of model-estimated total	Model estimate: total human rabies deaths	Model estimate: total human rabies exposures
2005	25%	0 (0%)	12	124	2439
2006	25%	0 (0%)	6	62	1153
2007	30%	0 (0%)	3	34	656
2008	30%	0 (0%)	2	23	467
2009	35%	0 (0%)	2	21	439
2010	0%	1 (4%)	3	28	722
2011	0%	13 (8%)	17	169	4619
2012	35%	2 (0.4%)	45	447	8901
2013	0%	0 (0%)	13	132	2331
2014	0%	0 (0%)	11	105	2316
**After the implementation of Haiti Animal Rabies Surveillance Program (HARSP) and PAHO Human Rabies Vaccine Training Program**					
2015	50%	5 (4%)	13	127	2402
2016	10%	7 (23%)	3	30	558
2017	0% (Ouest)<br>70% (Non-Ouest)	9 (24%)	4	37	881
2018	70% (Ouest)<br>0% (Non-Ouest)	4 (7%)	6	56	1185
2019	0% (Ouest)<br>70% (Non-Ouest)	2 (22%)	1	9	166
2020	0%	1 (7%)	2	15	453
**Overall (2005-2020)**		**44 (3%)**	**142**	**1,419**	**29,688**
Pre-HARSP (2005-2014)		16 (1%)	114	1,145	24,043
Post-HARSP (2015-2020)		28 (10%)	27	274	5,645

[1]Vaccination was assumed to begin halfway through the year. [2]Assuming 10% case detection (see Methods).

The three parameter sets used for sensitivity analyses showed similar trends over time through 2020, as can be shown in Figures S1-S3.

APPENDIX – SUPPLEMENTAL METHODS AND RESULTS

The urgency of resuming disrupted dog rabies vaccination campaigns: a modeling and cost-effectiveness analysis

Figure S1 shows the number of human rabies deaths per year from 2005-2020 estimated by the model for both Ouest Department and outside Ouest Department. These estimates are roughly consistent with the estimate of 130 human rabies deaths per year in Haiti (13).

Fig S1: Model estimates of human rabies deaths per year, 2005-2020

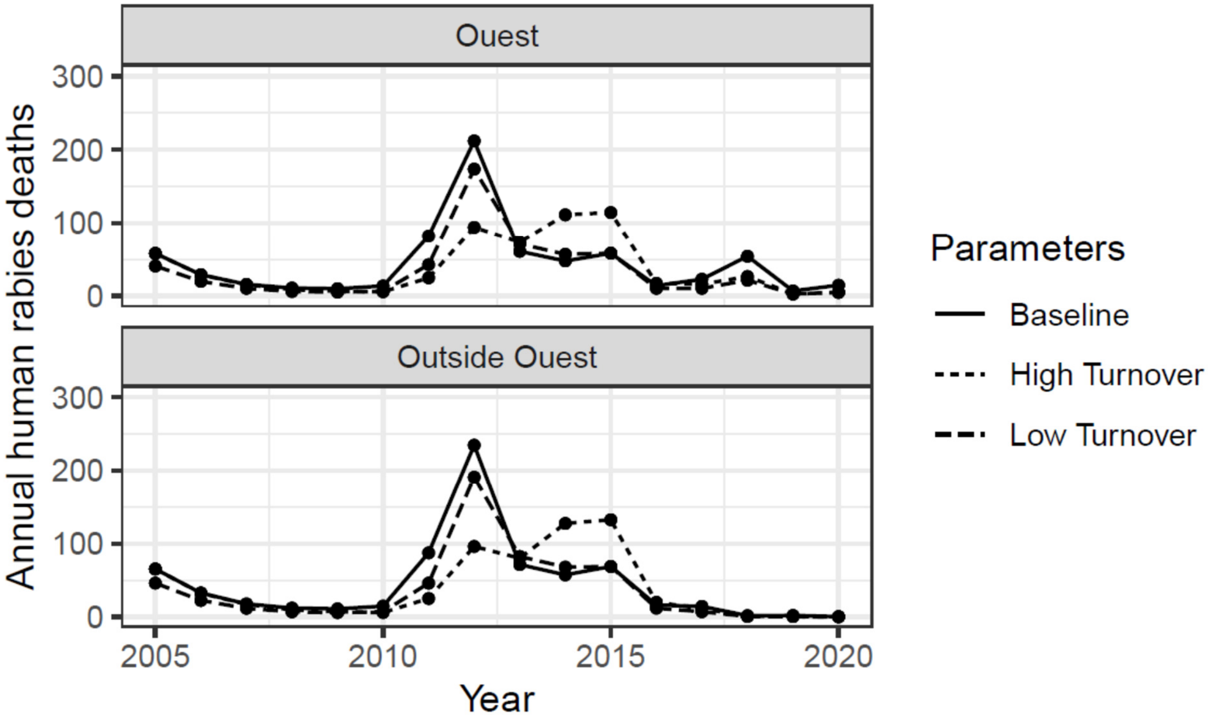

Figure S2 shows the number of human rabies exposures estimated by the model per year from 2005-2020 for both Ouest Department and outside of Ouest Department. Data from Haiti showed 1,111 total individuals initiated PEP during the first 17 weeks of 2016, including individuals with exposures to animals of unknown rabies status. Assuming 1111*52/17=3398 individuals initiated PEP during all of 2016, and comparing with the baseline modeled estimate of 558 true human rabies exposures, suggests a ratio of 6.1 PEP courses per true rabies exposure. Data from the first 48 weeks of 2020 showed 3566 initiated PEP. Assuming 3566*52/48=3863 individuals initiated PEP during all of 2020, and comparing with the baseline modeled estimates of 453 true human rabies exposures, suggests a ratio of 8.5 PEP courses per true rabies exposure.

APPENDIX – SUPPLEMENTAL METHODS AND RESULTS

The urgency of resuming disrupted dog rabies vaccination campaigns: a modeling and cost-effectiveness analysis

Fig S2: Model estimates of human rabies exposures per year, 2005-2020

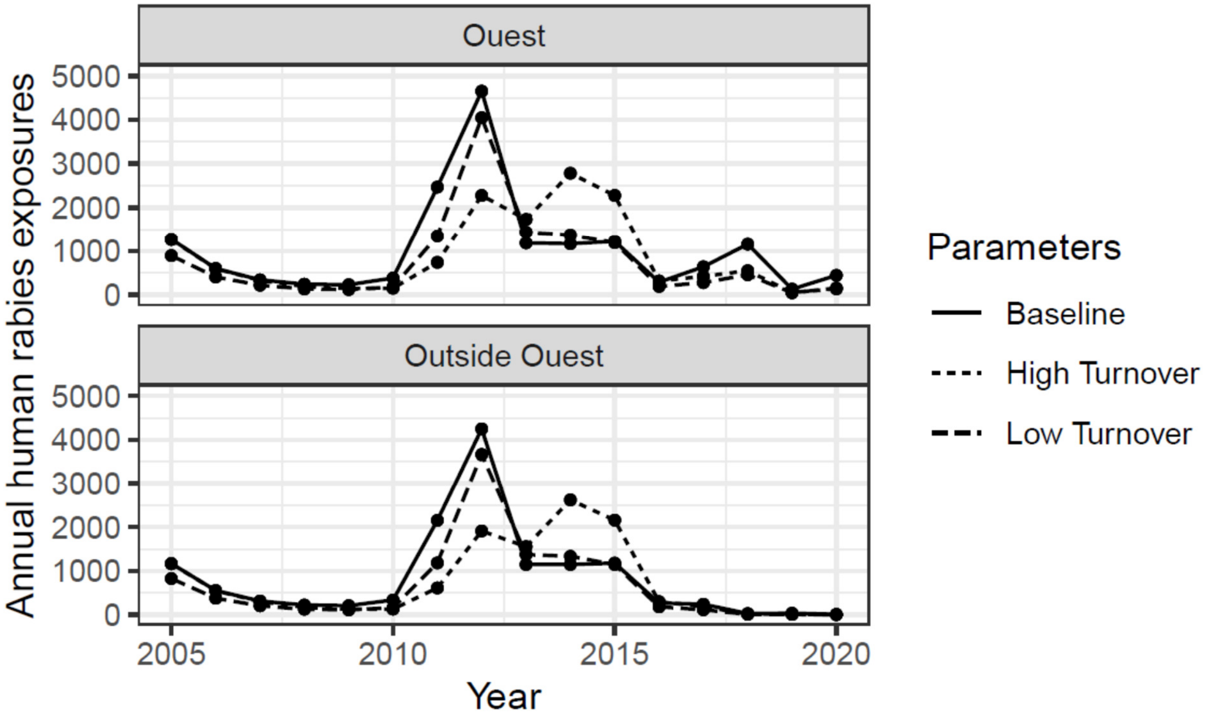

Figure S3 shows the number of canine rabies cases per year from 2005-2020 for both Ouest Department and outside of Ouest Department, divided by the size of the canine population at the end of the given year. This is not exactly incidence, considering that population size is measured at the end of the year, and the high turnover of the dog population throughout the year. Nevertheless, the estimates appear similar to data from other settings that generally report below 0.5-2% of dogs developing rabies per month, as summarized in (19). One previous study reported 10% of dogs found dead in Haiti were positive for rabies (20). This further validates that our model estimated total human rabies deaths and human exposure are within a reasonable range compared to the observed surveillance data and previously published studies.

The urgency of resuming disrupted dog rabies vaccination campaigns: a modeling and cost-effectiveness analysis

Fig S3: Model estimates of annual canine rabies cases divided by canine rabies population at year end, 2005-2020

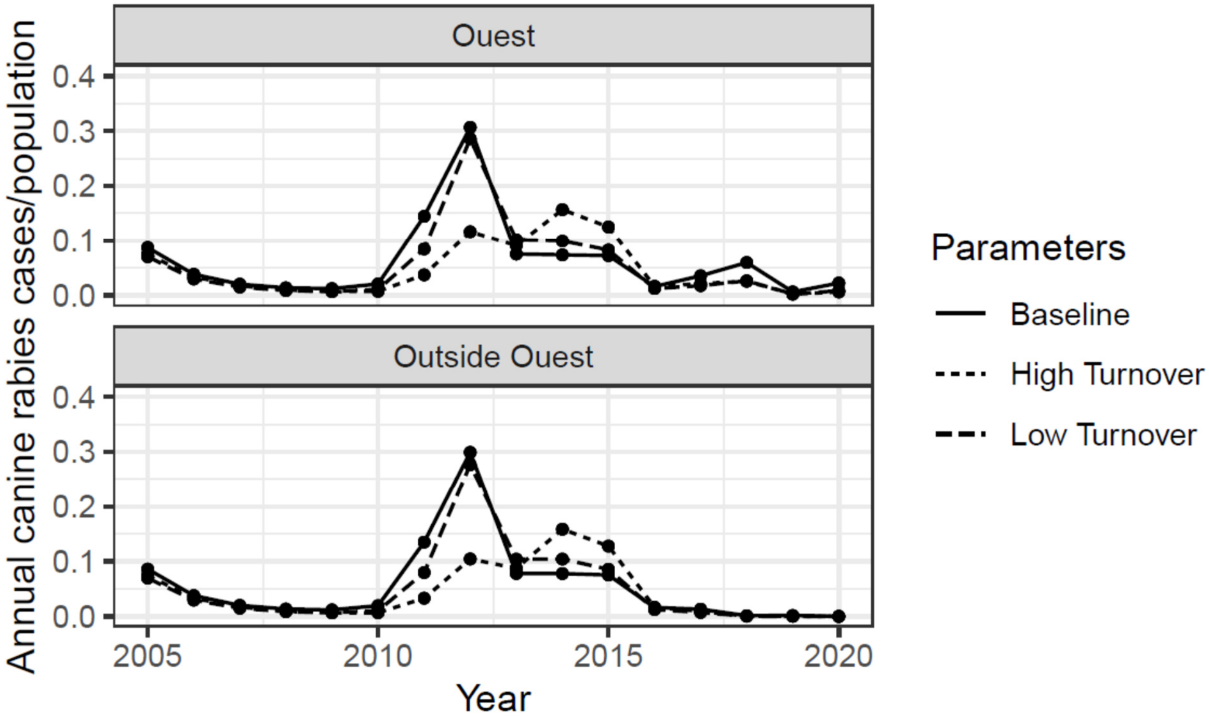

APPENDIX – SUPPLEMENTAL METHODS AND RESULTS

The urgency of resuming disrupted dog rabies vaccination campaigns: a modeling and cost-effectiveness analysis

Supplemental Results

Table S4 compares animal surveillance data with model estimates of rabid dogs. The estimated case detection is low: 0.1-0.6% in Ouest Department, compared to 0-3.9% outside Ouest Department. Investigations focus primarily on dog bites. The higher case detection rates outside Ouest might be explained in part by the higher number of rabies investigators outside Ouest Department (4 investigators in Ouest Department vs 33 outside, despite higher model estimates of rabid dogs in Ouest Department than outside for the time period depicted here).

Table S4: Surveillance data on suspected and confirmed rabid animals, 2018-2020

Time period	Investigations	Tested*	Confirmed	Percentage of investigations confirmed positive	Model estimated rabid dogs	Case detection rate (confirmed/model estimate)
**Ouest Department**						
2018 Fall	382	7	3	0.8%	2,003	0.1%
2019 Spring	401	5	3	0.7%	577	0.5%
2019 Fall	318	2	1	0.3%	604	0.2%
2020 Spring	292	3	1	0.3%	1,154	0.1%
2020 Fall[1]	284	11	8	2.8%	3,030	0.3%
**Outside Ouest Department**						
2018 Fall	946	13	7	0.7%	355	2.0%
2019 Spring	1,177	29	17	2.5%	667	2.6%
2019 Fall	886	8	6	0.7%	193	3.1%
2020 Spring	589	7	0	0%	77	0%
2020 Fall[1]	398	3	1	0.3%	114	0.9%

*Only investigated animals with high suspicion of rabies are tested.

[1]Data are included through November 19, 2020 only, but model output includes the entire 6-month period.

The urgency of resuming disrupted dog rabies vaccination campaigns: a modeling and cost-effectiveness analysis

Figure S4: Historical and projected canine rabies cases in Haiti, 2015-2025, and potential impact of delaying restarting dog rabies vaccination until either 2021 or 2022.

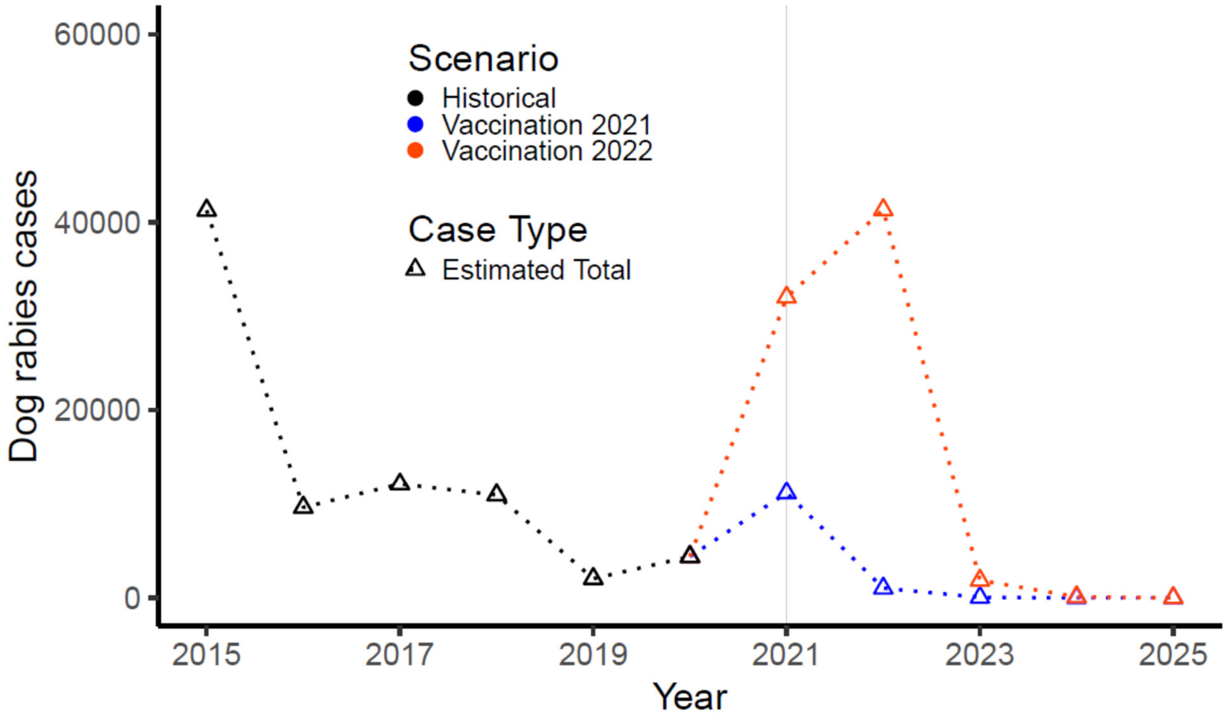

* Triangles (connected by dotted lines) represent model estimates of total dog rabies cases. Colors compare historical data and estimates for 2015-2020 (black) with model predictions if vaccination campaigns resume in 2021 (blue) or 2022 (orange). See Figure 2 in the main text for similar estimates of human rabies deaths.

APPENDIX – SUPPLEMENTAL METHODS AND RESULTS

The urgency of resuming disrupted dog rabies vaccination campaigns: a modeling and cost-effectiveness analysis

References

1. Borse RH, Atkins CY, Gambhir M, Undurraga EA, Blanton JD, Kahn EB, et al. Cost-effectiveness of dog rabies vaccination programs in East Africa. PLoS neglected tropical diseases. 2018;12(5):e0006490.
2. Jeon S, Cleaton J, Meltzer MI, Kahn EB, Pieracci EG, Blanton JD, et al. Determining the post-elimination level of vaccination needed to prevent re-establishment of dog rabies. PLoS neglected tropical diseases. 2019;13(12):e0007869.
3. The World Bank Data. Haiti [Available from: https://data.worldbank.org/country/HT.
4. Etheart MD, Kligerman M, Augustin PD, Blanton JD, Monroe B, Fleurinord L, et al. Effect of counselling on health-care-seeking behaviours and rabies vaccination adherence after dog bites in Haiti, 2014-15: a retrospective follow-up survey. The Lancet Global health. 2017;5(10):e1017-e25.
5. Ma X, Monroe BP, Cleaton JM, Orciari LA, Gigante CM, Kirby JD, et al. Public Veterinary Medicine: Public Health: Rabies surveillance in the United States during 2018. Journal of the American Veterinary Medical Association. 2020;256(2):195-208.
6. Mauti S, Traoré A, Hattendorf J, Schelling E, Wasniewski M, Schereffer JL, et al. Factors associated with dog rabies immunisation status in Bamako, Mali. Acta tropica. 2017;165:194-202.
7. Zinsstag J, Lechenne M, Laager M, Mindekem R, Naïssengar S, Oussiguéré A, et al. Vaccination of dogs in an African city interrupts rabies transmission and reduces human exposure. Science translational medicine. 2017;9(421).
8. Moore MC, Davis RD, Kang Q, Vahl CI, Wallace RM, Hanlon CA, et al. Comparison of anamnestic responses to rabies vaccination in dogs and cats with current and out-of-date vaccination status. Journal of the American Veterinary Medical Association. 2015;246(2):205-11.
9. Kitala P, McDermott J, Kyule M, Gathuma J, Perry B, Wandeler A. Dog ecology and demography information to support the planning of rabies control in Machakos District, Kenya. Acta tropica. 2001;78(3):217-30.
10. Schildecker S, Millien M, Blanton JD, Boone J, Emery A, Ludder F, et al. Dog Ecology and Barriers to Canine Rabies Control in the Republic of Haiti, 2014-2015. Transboundary and emerging diseases. 2017;64(5):1433-42.
11. Hampson K, Dushoff J, Cleaveland S, Haydon DT, Kaare M, Packer C, et al. Transmission dynamics and prospects for the elimination of canine rabies. PLoS biology. 2009;7(3):e53.
12. Kurosawa A, Tojinbara K, Kadowaki H, Hampson K, Yamada A, Makita K. The rise and fall of rabies in Japan: A quantitative history of rabies epidemics in Osaka Prefecture, 1914-1933. PLoS neglected tropical diseases. 2017;11(3):e0005435.
13. Hampson K, Coudeville L, Lembo T, Sambo M, Kieffer A, Attlan M, et al. Estimating the global burden of endemic canine rabies. PLoS neglected tropical diseases. 2015;9(4):e0003709.
14. Knobel DL, Cleaveland S, Coleman PG, Fèvre EM, Meltzer MI, Miranda ME, et al. Re-evaluating the burden of rabies in Africa and Asia. Bulletin of the World Health Organization. 2005;83(5):360-8.
15. Cleaveland S, Dye C. Maintenance of a microparasite infecting several host species: rabies in the Serengeti. Parasitology. 1995;111 Suppl:S33-47.
16. Weber EM, Seaman VY, Stewart RN, Bird TJ, Tatem AJ, McKee JJ, et al. Census-independent population mapping in northern Nigeria. Remote sensing of environment. 2018;204:786-98.
17. Facebook Connectivity Lab and Center for International Earth Science Information Network - CIESIN - Columbia University. High Resolution Settlement Layer (HRSL) 2016 [Available from: https://www.ciesin.columbia.edu/data/hrsl/#data.

18. Gibson AD, Handel IG, Shervell K, Roux T, Mayer D, Muyila S, et al. The Vaccination of 35,000 Dogs in 20 Working Days Using Combined Static Point and Door-to-Door Methods in Blantyre, Malawi. PLoS neglected tropical diseases. 2016;10(7):e0004824.
19. Morters MK, Restif O, Hampson K, Cleaveland S, Wood JL, Conlan AJ. Evidence-based control of canine rabies: a critical review of population density reduction. The Journal of animal ecology. 2013;82(1):6-14.
20. Wallace RM, Reses H, Franka R, Dilius P, Fenelon N, Orciari L, et al. Establishment of a High Canine Rabies Burden in Haiti through the Implementation of a Novel Surveillance Program [corrected]. PLoS neglected tropical diseases. 2015;9(11):e0004245.
